# Fatigue in chronic hepatitis B patients is significant and associates with autonomic dysfunction

**DOI:** 10.1186/s12955-019-1200-3

**Published:** 2019-07-25

**Authors:** Hong Wang, Ying Zhou, Rong Yan, Guo Qing Ru, Li Li Yu, Jiong Yao

**Affiliations:** 1 Department of Infectious Diseases, Zhejiang Provincial People’s Hospital, People’s Hospital Hang Zhou Medical College, 108, Shan Tang Road, Hangzhou, 310016 Zhejiang Province China; 2 Department of Pathology, Zhejiang Provincial People’s Hospital, People’s Hospital Hang Zhou Medical College, Zhejiang, China; 3 Department of Medical Record Statistic Information, Zhejiang Provincial People’s Hospital, People’s Hospital Hang Zhou Medical College, Zhejiang, China

**Keywords:** HBV, Fatigue, Autonomic, Symptom

## Abstract

**Background:**

Fatigue is an important clinical finding in patients with chronic hepatitis virus infection. However, studies assessing fatigue in patients with chronic hepatitis B (CHB) are very limited. This study aimed to quantify the severity of fatigue in patients with CHB, to determine whether perceived fatigue reflects impairment of functional ability, and to explore potential causes.

**Methods:**

A total of 133 patients with histologically proven CHB and 59 community controls were assessed using the fatigue impact scale (FIS).

**Results:**

The degree of fatigue was significantly higher in patients with CHB than in controls (mean (range) FIS 24.9 (0–91) vs. 15.7 (0–31), *p* < 0.001). Fatigue experienced by patients with CHB was similar to that in primary biliary cirrhosis (PBC) (*n* = 20) (FIS 22.2 vs. 20.9, *p* = 0.28). No association was found between FIS and biochemistry and histological parameters of liver disease severity. Significant associations were found between fatigue severity and cognitive impairment (*r* = 0.39, *p* < 0.001), daytime somnolence (*r* = 0.32, *p* < 0.001), scores of the Chronic Liver Disease Questionnaire (*r* = − 0.31, *p* < 0.001), and autonomic symptoms (*r* = 0.43, *p* < 0.001). The level of autonomic symptom was the only factor independently associated with the degree of fatigue.

**Conclusion:**

Fatigue is a significant problem of functional ability impairment in CHB and similar in degree to that in PBC patients. Fatigue in patients with CHB appears to be unrelated to the severity of liver disease but is associated with significant autonomic symptoms.

## Background

Chronic hepatitis B virus (CHB) infection is a public health problem. Patients with CHB have been reported to have an increased risk for cirrhosis and hepatocellular carcinoma development [1]. Hepatitis B virus (HBV) infection is one of the most common indications for liver transplantation worldwide [2]. This hepatic manifestation of HBV has a profound negative impact on patients’ health-related quality of life [3, 4]. It is now increasingly recognized that quality of life (QOL) can be significantly impaired in several chronic liver diseases (CLDs) through the impact of systemic features such as fatigue, non-encephalopathic cognitive impairment, and sleep disturbance [5, 6]. Although the impact of individual symptoms such as fatigue is undoubted in CLD patients, their measurement in isolation is reductionist in philosophy and can underestimate what matters most to patients. The ultimate goal of such clinical status is to be able to lead a normal life, and assessment approaches are now well described that can formally include, in a reproducible fashion, functional status.

Objective studies using fatigue assessment tools such as the fatigue impact scale (FIS) and the fatigue severity scale (FSS) have confirmed that patients with CLD have significant fatigue [7, 8]. A number of studies reported that fatigue had significant high prevalence in CLD patients with several etiological groups [9, 10]. For example, study showed that patients with primary biliary cirrhosis (PBC) experience a wide range of symptoms that can impact significantly on their quality of life (QOL). The most prominent of these symptoms in many patient populations with PBC is fatigue [10]. Fatigue is being recognized as a major factor that impacts the QOL in association with CLD. Moreover, fatigue has been particularly associated with the presence of autonomic dysfunction and excessive sleepiness. This impairment in functional ability is unrelated to markers of liver disease severity such as bilirubin and albumin [11, 12].

However, studies assessing fatigue in patients with CHB are very limited [13, 14]. Furthermore, quantitative evidence is lacking as to what degree this perception is true and what the associated factors are. To date, no study has considered overall function as an outcome following chronic HBV infection. Because of the significant impact of fatigue in patients with CHB, addressing the issue of the extent to which they can reduce functional ability and the development and application of approaches that can improve function is necessary. Hence, this study aimed to examine the severity of fatigue in a sequential cohort of Chinese patients with CHB with liver biopsy results and compare it with community controls. We investigated whether fatigue persists in patients with CHB and explored the relationship between fatigue and function to determine where interventions might be targeted to improve fatigue.

## Patients and methods

### Study design

A cross-sectional comparative study was performed in two phases. Phase 1: Fatigue was compared between a group of patients with CHB who had liver biopsy and community controls. It was examined in a subgroup of the CHB group matched for age and sex with primary biliary cirrhosis (PBC) group. Phase 2: Fatigue and its associations in CHB were examined.

### Patients and control groups

CHB patients: All patients who underwent liver biopsy at the liver center of Zhejiang Provincial People’s Hospital between January 2015 and June 2017 were invited to complete a series of functional assessment and symptom quantification tools. All the patients who fulfilled the following inclusion criteria were enrolled: age ≥ 18 years, diagnosis of CHB based on HBsAg positivity for > 6 months, detectable HBV-DNA level ≥ 10^5^ copies/mL for HBeAg-negative patients and ≥ 10^4^ copies/mL for HBeAg-positive patients, and no previous or concomitant anti-HBV therapy. Patients with liver comorbidities, such as hepatitis delta superinfection, hepatitis C virus co-infection, chronic alcohol consumption (< 30 g of pure alcohol per day), Wilson disease, human immunodeficiency virus co-infection, and cirrhosis with encephalopathy, and patients with depressive symptoms and auto-immune hepatitis were excluded.

Community-dwelling group: The patient group was matched group-wise on age -, education-, and gender-matched basis with normal community controls who had completed the fatigue assessment tool. However, normal community controls did not undergo liver function tests as a part of screening.

PBC group: This chronic liver disease control group classically associated with fatigue was also matched for age and sex. Twenty of the total group proved possible to match from the PBC control group.

No selection was made with regard to co-morbidity, fatigue status, or function ability in any of the study groups. This study was conducted in compliance with the ethical guidelines of the 1975 Declaration of Helsinki and was approved by the ethical committee at Zhe Jiang Provincial People’s Hospital. All patients signed an informed consent prior to screening.

### Assessment tools

Five functional and symptom assessment tools were completed by patients with CHB on the day prior to liver biopsy. The following were the assessment tools and their rationale for inclusion:

#### Chronic liver disease questionnaire (CLDQ) [15]

CLDQ is a liver-specific tool that assessed the QOL in patients with CHB infection by measuring the functional ability of the patients. The CLDQ is comprised of 29 items in the following domains: abdominal symptoms, fatigue, systemic symptoms, activity, emotional function, and worry. Patients were instructed to rate their ability to carry out daily activity ties on a seven-point scale: “7, none of time ,” to “1, all of the time.” All scores of the seven domains are added together (range 29–203 from worst to best QOL). Lower scores indicate worse quality of life and therefore greater functional impairment.

#### Cognitive failures questionnaire (CFQ) [16, 17]

CFQ was used to determine whether patients with CHB experienced cognitive symptoms more frequently than the matched controls, indicating worse cognitive impairment. The patients and controls completed the CFQ, a fully validated tool that assesses the level of cognitive ability. The CFQ assesses the prevalence of cognitive symptoms by measuring the frequency of cognitive slips or failures occurring in everyday life. The cognitive abilities assessed in the CFQ include memory, attention, concentration, forgetfulness, word-finding abilities, and confusion. The questionnaire consists of 25 items covering failure in perception, memory, and motor function and asks patients to rate how often these failures occur, on a 5-point scale of 0–4 (0 = never, 4 = very often). The responses for the 25 questions are added together to obtain the total CFQ score. The higher the score, the greater the cognitive impairment.

#### Fatigue impact scale (FIS) [18]

Studies have previously reported that fatigue is a significant factor in life quality impairment in patients with chronic liver disease. FIS assesses patient’s perception of how fatigue affects their cognitive, physical, and psychosocial functions. This includes the impact of fatigue on their work, family and financial responsibilities, their mood, their reliance on others, their social activities, and on their QOL. FIS is a 40-item scale, and subjects must rate how badly affected these items are because of fatigue on a 5-point scale ranging from 0 (no problem) to 4 (extreme problem). The total FIS score is calculated by adding all answers from the 40 questions (possible range 0–160). High scores indicate great impact of fatigue.

#### Orthostatic grading scale (OGS) [19]

The OGS is a self-report assessment tool consisting of five items, which assess the frequency of orthostatic symptoms, severity of orthostatic symptoms, condition under which orthostatic symptom occurs, activities of daily living, and standing time. Patients are asked to grade each item on a scale of 0–4(0 being the lowest and 4 the highest). The total OGS score is calculated by adding up the scores from each item. Higher scores indicate greater severity of autonomic dysfunction.

#### Epworth sleeping scale (ESS) [20]

Sleep disturbance is recognized as a factor in chronic liver disease. The Epworth sleeping scale is used to assess daytime hypersomnolence, a score of 10 or more being indicative of significant daytime hypersomnolence.

### Liver biopsy and serum biochemistry

All patients underwent percutaneous liver biopsy guided by ultrasonography. Liver biopsy was performed using 16-G Tru-Cut biopsy needles (Bard, Covington, GA, USA). The specimens were fixed, paraffin-embedded, and stained with hematoxylin and eosin. A minimum of 1.5 cm of liver tissue with at least 9 portal tracts was required for appropriate diagnosis. All liver biopsies were reviewed by one pathologist (G.Q.R), who had no knowledge of the clinical characteristics of the study subjects. Liver fibrosis was evaluated according to METAVIR scoring system.

Serum samples of the cohort were obtained on the day before liver biopsy. Biochemical tests for fasting plasma glucose, total cholesterol, triglycerides, alanine aminotransferase, aspartate aminotransferase, alkaline phosphatase, gamma-glutamyl transferase, bilirubin, albumin, and complete blood count were performed in the hospital’s clinical laboratory using commercially available assays. Hepatitis antibodies HBsAg, HBsAb HBeAg, HBeAb, HBcAb, and anti-HCV were measured using Clinical Laboratory Improvement Act (CLIA)-approved systems compatible with AASLD practice guidelines. Serum HBV-DNA levels were assessed using a real-time polymerase chain reaction system (ABI7300; 55 Applied Biosystems, CA, USA). The lower limit of detection was set at 200 copies/mL, and the linearity range was set from 200 to 20,000 copies/mL. Liver fibrosis was evaluated according to the METAVIR scoring system [15] as follows: F0, no portal fibrosis; F1 pericellular/perivenular or periportal fibrosis, F2, portal fibrosis with rare septa; F3, numerous septa or lobular distortion without cirrhosis; and F4, cirrhosis.

### Statistical analysis

Quantitative variables are expressed as means (standard deviation) if the distribution was normal or medians (interquartile range) otherwise. Categorical data are presented as frequencies and percentages. To determine whether the degree of functional impairment experienced by patients with CHB was influenced by the symptoms they experienced, we explored the univariate relationship among functional capacity and the symptom assessment tools of cognitive symptoms, fatigue, and autonomic dysfunction. Univariate analysis was performed by correlations using Spearman and Pearson ‘s tests. To determine whether the relationships between functional ability and the symptoms experienced by patients with CHB (cognitive impairment, fatigue, and autonomic dysfunction) are independent of each other, a multivariate analysis was performed using the logistic regression test. Differences in proportion were determined using chi-square tests. *P*-values < 0.05 were considered statistically significant. Statistical analyses were performed using SPSS version 20.0 software (IBM Corp., Armonk, NY USA).

## Results

### Phase 1: fatigue status in patients with CHB compared with the control and PBC groups

#### Clinical and demographic data

**Fig. 1:**
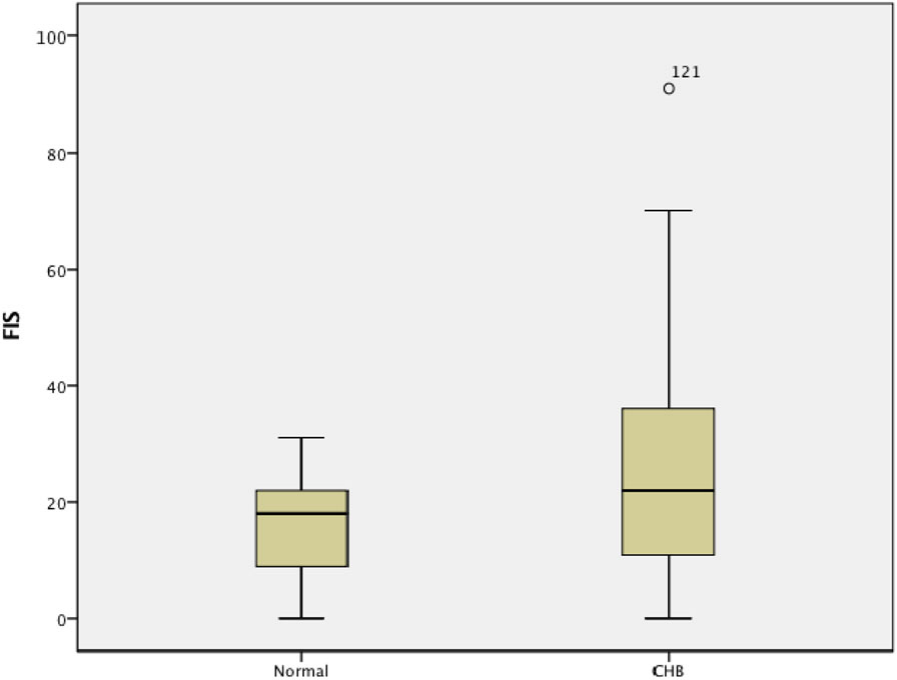
Fatigue (assessed using the fatigue impact scale) is significantly higher in chronic hepatitis B (CHB) group compared with age- and sex-matched controls, *p* < 0.001

A total of 138 patients with CHB and 49 controls fulfilled the inclusion/exclusion criteria and agreed to participate in the study. Patients were excluded if they had a Beck score consistent with depression. Five patients were excluded because of depression. All patients and controls completed chronic fatigue screener. One hundred thirty-three patients with HBV had additional testing for immunologic markers and liver histologic severity and completed CLDQ, CFQ, OGS, and ESS questionnaires. Table 1 summarizes the demographic and clinical characteristics of all patients infected with HBV. Compared with controls, fatigue severity was significantly higher in the CHB group (mean (range) 24.9 (0–91) vs. 15.7 (0–31); *p* < 0.01)) (Fig. 1).

**Table 1:** Characteristic of the chronic hepatitis B group and the normal control

	CHB patients	Controls		*p* value
n	133	49		
Age	35.6 ± 10.1	34.6 ± 12.9		0.16
Female(%)	48 (36.1)	24 (49)		
Albumin(g/l)	45.1 ± 4.0	–		
Billrubin (umol/dL)	16 ± 8.5	–		
Log_10_HBV DNA(×copies/ML)	6.5 ± 3.1	–		
ALT(U/L)	48.5 (8–373)	–		
AST(U/L)	37.1 (11–214)	–		
BMI	24.3 ± 4.4	23.2 ± 5.3		0.24
OGS	1.8 (2.3–5.4)	–		
FIS	24.9 (0–91)	15.7 (0–31)		0.005
CLDQ	58.7 ± 18.8	–		
COG-FAIL	23.1 ± 12.7	–		
ESS	5.5 ± 3.64	–		

*ALT* alanine transaminase, *AST* aspartate aminotransferase, *BMI* body mass index, *ESS* Epworth Sleepiness Scale, *FIS* Fatigue Impact Scale, *CLDQ* Chronic Liver Disease Questionnaire, *OGS* Orthostatic Grading Scale. Continuous variables are presented as mean and standard deviation; categorical variables are expressed as counts and medians

#### Fatigue status in patients with CHB compared with PBC

To compare the prevalence of fatigue between patients with CHB and patients with PBC, the data from 20 patients with CHB were compared with those of 20 matched patients with PBC. No significant differences were observed in FIS and CLDQ scores between patients with CHB and PBC (Table 2).

**Table 2:** Comparison fatigue between CHB patients and PBC patients

	CHB	PBC	*p* value
N	20	20	
Age	30.9 ± 10.6	34.3 ± 10.1	0.23
FIS	22.2 ± 12.9	20.9 ± 9.2	0.28
CLDQ	56.8 ± 19.4	57.6 ± 26.3	0.9

*FIS* Fatigue Impact scale, *CLDQ* Chronic Liver Disease Questionnaire

### Phase 2: fatigue and its associations in patients with CHB

**Fig. 2:**
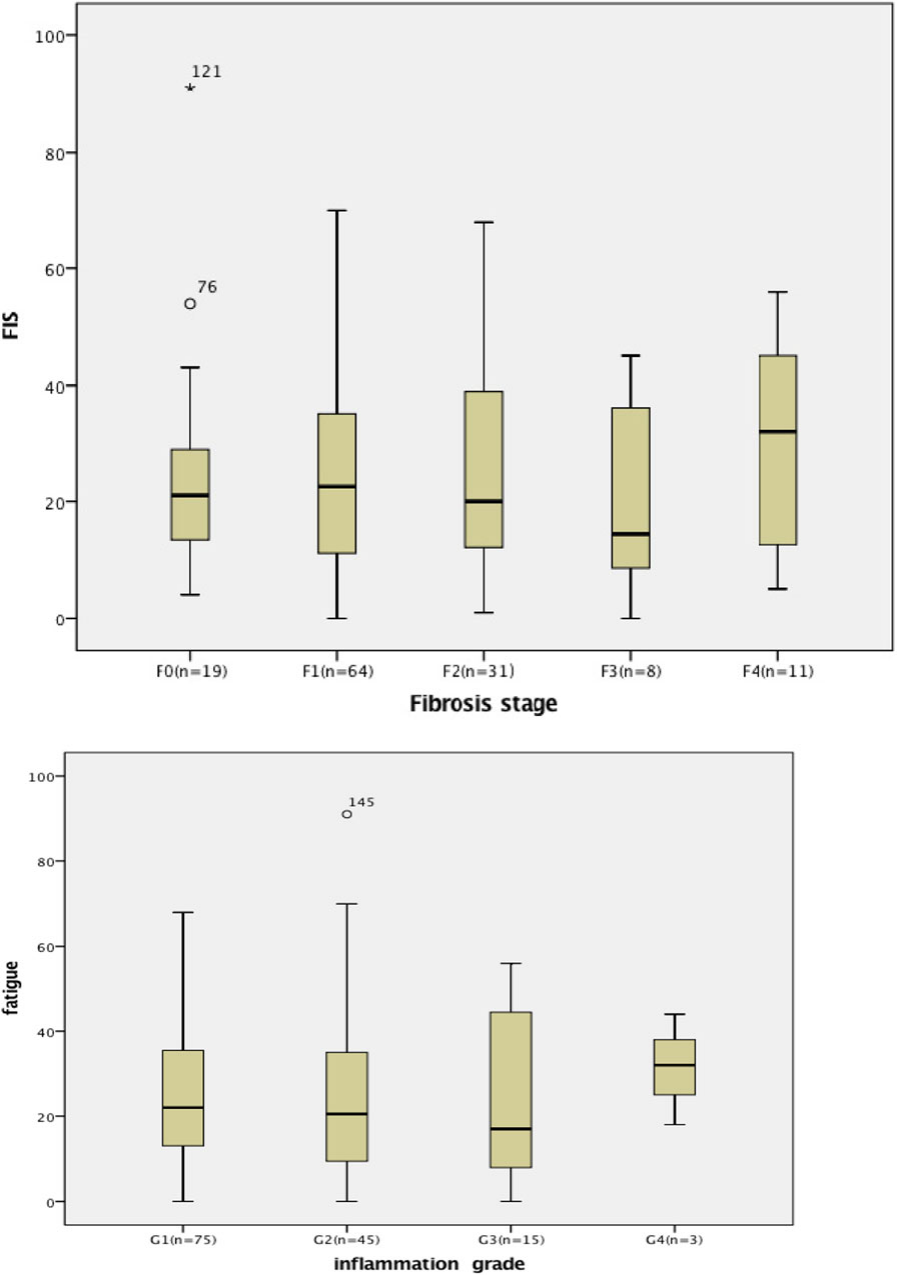
Fatigue severity is unrelated to liver disease severity measured as fibrosis stage and inflammation grade

**Fig. 3:**
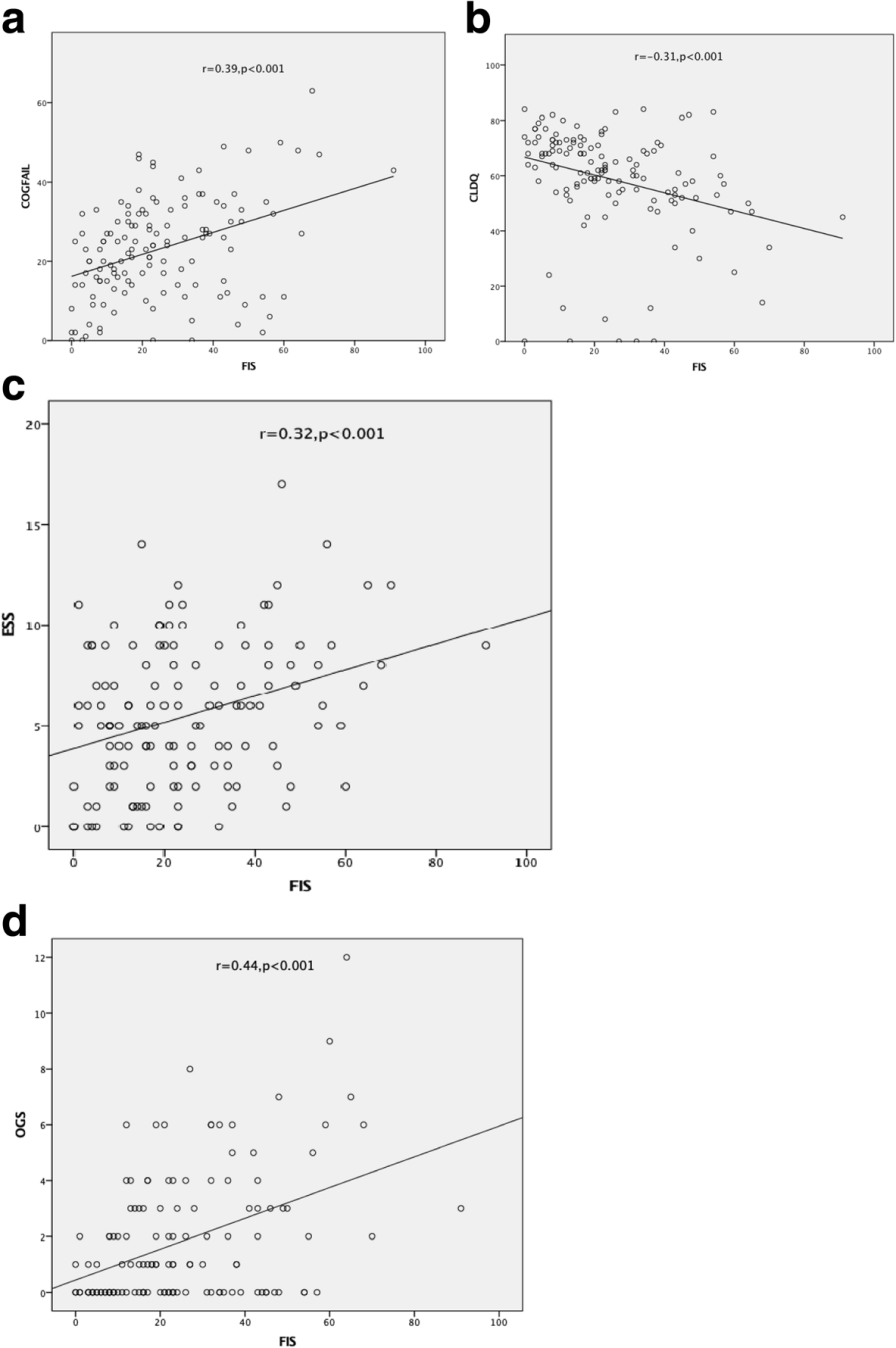
Correlation between fatigue severity (assessed using the fatigue impact scale) and (**a**) cognitive symptoms (assessed by COG-FAIL), (**b**) quality of life as assessed by Chronic Liver Disease Questionnaire (CLDQ), (**c**) daytime somnolence as assessed by Epworth Sleepiness Scale (ESS), (**d**) orthostatic dysfunction symptoms, as assessed by Orthostatic Grading Scale (OGS) in patients with CHB

To seek evidence of potential mechanisms of fatigue in patients CHB, we looked for correlations between fatigue status and clinical, biochemical, and histological variables in the cohort of patients with CHB. No relationship was found between FIS and liver biochemical markers (Table 3)**.** Similarly, no statistical difference was observed in FIS scores according to the severity of liver histology (Fig. 2). In contrast, elevation in FIS was strongly associated with the levels of systemic symptoms related to scores of CLDQ, cognitive impairment, daytime somnolence, and autonomic symptoms (Fig. 3).

**Table 3:** The relationship of biochemical statue with FIS in CHB patients

	Correlation with FIS	
Variables	r	*p* value
Age (years)	−0.016	0.86
BMI	0.069	0.43
Albumin(g/L)	−0.06	0.49
ALT(U/L)	0.06	0.51
AST(U/L)	0.06	0.5
Bilirubin (umol/L)	0.085	0.33
HBVDNA (copies/dL)	−0.09	0.3
PLT(×10^9^/L)	0.06	0.47

*ALT* alanine transaminase, *AST* aspartate aminotransferase, *BMI* body mass index, *PLT* Platelet

A multivariate model was then developed to determine what factors independently predicted fatigue in patients with CHB. A regression model was developed that included parameters considered important including age, autonomic symptoms (OGS), cognitive symptoms (COG-FAIL), and daytime somnolence (ESS). The only factor independently associated with the degree of fatigue was OGS (Table 4). No correlation was found between OGS and any biochemical or histological parameter of liver disease severity, HBV-DNA levels, body mass index, age, and sex. To explore this more fully and determine whether elevated OGS or FIS or both were influenced by liver disease severity, the groups were separated into those with excessive autonomic symptoms (OGS > 1.8), with and without significant fatigue (the mean FIS values from the normal control group, i.e., FIS ≥25), and potential difference between the groups was explored (Table 5). No significant difference was found between the CHB subgroups.

**Table 4:** Predictors of fatigue in the chronic hepatitis B patients

Variables	Wald	Sig.	Exp(B)	95% C.I.for EXP(B)	
				Lower	Upper
Age (years)	0.53	0.46	0.92	0.74	1.03
HBVDNA (copies/ml)	0.20	0.65	1	1	1
Inflammation Grade	0.03	0.85	1.07	0.53	2.18
COG-FAIL	1.13	0.58	0.96	0.84	1.10
ESS	3.66	0.41	1.26	0.72	2.20
OGS	9.38	0.002	1.39	1.13	1.72
Fibrosis stage	0.60	0.44	1.22	0.74	2.01
Constant	0.29	0.591	8.19		

*ESS* Epworth Sleepiness Scale, *OGS* Orthostatic Grading Scale

**Table 5:** Comparing those CHB patients with excessive orthostatic symptoms (OGS > 1.8) and fatigue (FIS ≥ 25) with those without

	OGS < 1.8		OGS > 1.8	
	FIS < 25	FIS > 25	FIS < 25	FIS > 25
n	39	39	15	40
Female(%)	21 (26.9)	5 (6.4)	12 (20)	10 (40)
Age (years)	36.5 ± 10.2	37.1 ± 11.2	35.2 ± 8.5	33.310.6
BMI (kg/m2)	24.3 ± 4.1	25.8 ± 5.5	23.5 ± 4.2	23.8 ± 3.9
Albumin(g/l)	45.5 ± 4.6	44.9 ± 3.8	45.4 ± 2.9	44.2 ± 3.2
ALT(U/L)	54.9 ± 48.7	37.9 ± 19.9	30.2 ± 16.1	66.2 ± 57.1
AST(U/L)	40.3 ± 26.2	31.2 ± 13.7	27.1 ± 8.6	42.9 ± 39.8
BiL (umol/L)	16.5 ± 10.8	15.6 ± 5.2	17.4 ± 9.2	14.5 ± 9.8
Log_10_HBVDNA(copies/dl)	9.7 ± 0.5	4.5 ± 0.1	2.4 ± 0.4	5.2 ± 0.1
Inflammation Grade(G1/G2/G3/G4)	28/17/9/1	13/7/1/2	15/8/0/0	20/19/8/5
Fibrosis stage(F0/F1/F2/F3/F4)	8/28/10/4/5	3/9/5/2/4	3/9/10/1/0	5/18/6/1/2

*ALT* alanine aminotransferase, *AST* aspartate aminotransferase, *BMI* body mass index. None of the comparisons is significant (by analysis of variance)

## Discussion

In this study, we have shown that the degree of fatigue experienced by a cohort of treatment-naive patients with CHB is significantly greater than that experienced by age- and sex-matched community controls. We have used broad-based and widely accepted tools of functional status in patients with CHB with liver biopsy results. Our finding suggests that fatigue is far from being normal and is impaired across all domains. Furthermore, the fatigue experienced by patients with CHB was not associated with parameters suggestive of live dysfunction, HBV virus loads, or the severity of liver histology but rather with those systemic symptoms increasingly recognized in other chronic liver diseases, notably cognitive symptoms and autonomic symptoms. The rationale behind the study was to identify the true impact of the disease on patients’ lives, to identify factors responsible for any impairment in function ability, and to identify those targets for intervention which are most likely to result in improvement in the quality of life.

To confirm whether fatigue is a problem in CHB, we compared our results with those of another fatigue-associated liver disease, PBC. No difference was observed in fatigue severity between the two groups. The result indicated that CHB patients might also experience significant fatigue which is undoubted in PBC. There are three important elements for this study that added to our understanding of the functional symptoms and impact of fatigue in CHB. First, the disease is associated with fatigue, indicating that the functional ability of patients is impaired to a greater degree than what has been previously shown. Second, the major determinants responsible for both functional impairment and the specific symptoms contributing to it seem to be the fatigue and the autonomic dysfunction (manifest through elevation in FIS and OGS scores). Third, fatigue is independently associated with autonomic dysfunction. The importance of this finding is that autonomic dysfunction is potentially reversible making this a key potential target for intervention if we wish to decrease the degree of fatigue and thus improve the QOL in patients with CHB. This study, therefore, has potential implications for individual patients with CHB, and we might look to improve their treatment.

Liver disease severity appears not to be directly linked to fatigue severity. However, no patients seen in the current study with end-stage disease may conceivably experience fatigue resulting from hepatic encephalopathy. Liver disease severity, as measured by conventional biochemistry markers and liver histology parameters was not entirely unrelated to FIS in patients with CHB. This is in agreement with the findings of previous studies performed on different etiological samples of chronic liver disease [21–23]. The finding of no association between liver histological parameters and fatigue severity would suggest that the results from this study would applicable to patients with CHB seen in routine practice. These findings have implication for clinical management of patients with CHB as they would suggest that improving the liver abnormally may not impact significantly on the QOL in general and fatigue in particular. The finding of a relationship between autonomic dysfunction and fatigue led to several important conclusions. First, in the most profoundly affected group of patients in whom serious fatigue is present, autonomic dysfunction is present almost universally. It would suggest that intervention to correct autonomic dysfunction could be of some clinical benefit to patients with CHB and is worthy of future study. Second, a significant group of patients with CHB (of those patients experiencing mild or medium fatigue) do not show significant autonomic dysfunction. This finding would suggest that these features are not automatic associations of the disease process but represent an additional process associated with CHB, ultimately independent of it. This raises interesting questions regarding the additional risk factors that must be present in patients who experience fatigue. Third, specific studies are needed in CHB to examine whether the treatments such as tilt training and volume expanders are effective at reducing orthostatic symptoms and whether this is, in turn, associated with improvements in fatigue and ultimately QOL.

The observation that a significant proportion of patients with CHB are not troubled by fatigue raises important questions regarding the cause of this symptom. Depression is a well-known cause of fatigue [24, 25]. However, in this study, patients with depression had been excluded from the study, which indicated that it might be another reason that contributed to fatigue in patients with CHB. Although the pathophysiological mechanisms of fatigue remain unclear, there may be both central and peripheral effects. Depression can clearly be a cause of central fatigue [20]. However, in this study, the depression factor had been ruled out; it is likely that fatigue in HBV has more in common mechanistically with other etiologies of CLDs. Based on a previous study on HCV, central fatigue is thought to occur as a consequence of combined inflammatory and neurochemical factors [26, 27], and chronic hepatitis C infection may attribute to neuropsychological, neurophysiological, and cerebral ^1^H magnetic resonance abnormalities. We raised a hypothesis that a central fatigue model may also exist in patients with CHB arising as a consequence of inflammatory processes during liver chronic inflammation. Whether cerebral ^1^ H magnetic resonance abnormalities are present in patients with CHB needs further study. Although fatigue was the symptom with the highest overall load in CHB, the symptoms set with the greater impact were autonomic symptoms. The etiology of autonomic dysfunction in CHB remains unclear. It perhaps arises as a consequence of inflammatory processes occurring as a result of chronic liver inflammation; it would affect autonomic areas of the brain, thus leading to secondary peripheral autonomic effects [28, 29].

This study has several limitations. The size and characteristics of this consecutive clinical sample restrict the generalization of results. All subjects in our study were recruited from one tertiary liver care center, potentially introducing problems with external validity of our results to patients seen in the other care centers. Secondly, it is possible that only CHB patients who might worry about their health status would undergo liver biopsy. Therefore, results of this study may be affected by selection bias. In addition, use of the convenient samples and a relatively small sample size of 133 might influence on the generalization of the study. It would need external validity.. Another limitation is that it is clearly a cross-sectional study, and performing a longitudinal study would be of great value in exploring fatigue over time in this group. However, the fact that fatigue was found in those with early liver disease suggests that a recruitment bias does not account for these findings.

## Conclusions

In conclusion, we have shown that fatigue is a true and specific feature of chronic hepatitis B and one that can adversely affect QOL. It does not, however, affect all patients, nor does it appear to simply result from the presence of advanced disease. A significant proportion of fatigue in CHB is associated with the presence of autonomic dysfunction. Potential interventions for these processes such as fluid management, salt intake management, and the use of midodrine are worth studying in the context of patients with CHB with fatigue.

## Data Availability

The datasets used and/or analyzed during the current study are available from the corresponding author on reasonable request.

## Abbreviations

ALT: Alanine aminotransferase
AST: Aspartate aminotransferase
CFQ: Cognitive Failures Questionnaire
CHB: Chronic HBV-infection
CLDQ: Chronic Liver Disease Questionnaire
DNA: Deoxyribonucleic acid
ESS: Epworth Sleeping Scale
FIS: Fatigue Impact Scale
HBeAg: HBV e antigen
HBsAg: HBV surface antigen
HBV: Hepatitis B virus
HCC: Hepatocellular carcinoma
HRQOL: Health-related quality of life
OGS: Orthostatic Grading Scale
PBC: Primary biliary cirrhosis
